# Mechanical Properties, Short Time Creep, and Fatigue of an Austenitic Steel

**DOI:** 10.3390/ma9040298

**Published:** 2016-04-20

**Authors:** Josip Brnic, Goran Turkalj, Marko Canadija, Domagoj Lanc, Sanjin Krscanski, Marino Brcic, Qiang Li, Jitai Niu

**Affiliations:** 1 Department of Engineering Mechanics, Faculty of Engineering, University of Rijeka, Rijeka 51000, Croatia; turkalj@riteh.hr (G.T.); markoc@riteh.hr (M.C.); dlanc@riteh.hr (D.L.); sanjink@riteh.hr (S.K.); mbrcic@riteh.hr (M.B.); 2 School of Material Science and Engineering, Henan Polytechnic University, Jiaozuo 454003, China; liqiangdoc@163.com (Q.L.); niujitai@163.com (J.N.); 3 School of Material Science and Engineering, Harbin Institute of Technology, Harbin 150001, China

**Keywords:** mechanical properties, creep test, fatigue, austenitic stainless steel

## Abstract

The correct choice of a material in the process of structural design is the most important task. This study deals with determining and analyzing the mechanical properties of the material, and the material resistance to short-time creep and fatigue. The material under consideration in this investigation is austenitic stainless steel X6CrNiTi18-10. The results presenting ultimate tensile strength and 0.2 offset yield strength at room and elevated temperatures are displayed in the form of engineering stress-strain diagrams. Besides, the creep behavior of the steel is presented in the form of creep curves. The material is consequently considered to be creep resistant at temperatures of 400 °C and 500 °C when subjected to a stress which is less than 0.9 of the yield strength at the mentioned temperatures. Even when the applied stress at a temperature of 600 °C is less than 0.5 of the yield strength, the steel may be considered as resistant to creep. Cyclic tensile fatigue tests were carried out at stress ratio *R* = 0.25 using a servo-pulser machine and the results were recorded. The analysis shows that the stress level of 434.33 MPa can be adopted as a fatigue limit. The impact energy was also determined and the fracture toughness assessed.

## 1. Introduction

The structure is usually designed both in accordance with the purpose for the use intended and with favorable material selection. Service life conditions are determined by the purpose of the structure and accordingly the properties of the material have to comply with these conditions. It is known that material properties are tied in with the material chemical composition, the processing path, and the resulting microstructure. The properties depending on the microstructure are called structure–sensitive properties, for example, yield strength, hardness, *etc.*, [1]. A material selection process for structural design includes issues such as strength, stiffness, weight, *etc.*, as mentioned in Ref. [2]. The designing process has to include an efficient selection of materials and it is conducted under the assumption that the engineering material does not contain any failures. Furthermore, the proper use of the structure ensures that no failure will occur in it during its service life [3]. Any engineering structure (or structural element) needs to be shaped. This means that the material is subjected to processes (called manufacture) that include forming (*i.e.*, forging, casting), joining (*i.e*., welding), material removal (*i.e.*, machining) and the finishing process [4]. In all the applied processes mentioned, *i.e.*, in designing, manufacturing, service life conditions and maintenance, various failure modes may occur. In engineering practice, many failures can occur and any failure has a cause of its origin and the mode of its representation. The analysis of failure provides an answer to how and why an engineering component has failed, and in this sense such a discipline becomes a very powerful tool in modern structural design [5]. Common causes of failures are usually mentioned such as pre-existing defects (for example pre-existing cracks), defects initiating from imperfections, but also categories such as design errors, misuse, inadequate maintenance, assembly error, *etc.* In engineering practice a lot of failure modes have been observed, for example, force induced elastic deformation, creep, yielding, buckling, fatigue, fracture, corrosion, thermal shock, *etc.*, [6]. Creep is mentioned as one of the possible failure modes and in this investigation the short-time creep resistance of the considered material will be examined. Creep as a thermally activated phenomenon is usually defined as time dependent behavior of the material where at constant stress (load) the strain continuously increases [7]. Its occurrence is appreciable at temperatures above 0.4 Tm, where Tm is the melting temperature [8]. This behavior of the metallic material is usually displayed in the form of a curve consisting of three stages (I-transient creep, II-steady–state creep, III-accelerating creep). Material properties, the features of its machinability as well as numerical analysis of the structure made of the considered material belong to the most important information about both material and structure. In this sense, it is also recommended to get an insight into some investigations not only made by the authors of this work but also by other authors [9,10,11,12,13,14,15,16,17,18,19,20,21,22,23]. The possibility of the use of the finite element method in the shear stress analysis of the structure made of any of the mentioned materials is presented in [24]. The main task of the research presented in this paper is to determine the properties of the materials so as to provide the relevant information about the structure and behavior of the material under certain conditions of exploitation.

In addition, a brief overview of recently published papers relating to steel X6CrNiTi18-10 is presented. The material damage and the microhardness variations of X6CrNiTi18-10 were analyzed in order to determine the incubation period, performing the tests at different cavitation conditions in a cavitation chamber [25]. Since the low-cycle fatigue damage evolution in the metastable austenitic steel causes a deformation induced phase transformation from austenite to martensite, the microstructure changes in the pre-crack stage were investigated [26]. In Ref. [27] the surface integrity of some materials, including X6CrNiTi18-10, was quantified by means of 2D and 3D surface roughness parameters, strain-hardening effects and associated residual stresses. Furthermore, the phase formation and annealing behavior are described after nitrogen PIII (Plasma immersion ion implantation) in two austenitic stainless steels, 1.4541 (X6CrNiTi18-10) and 1.4571 (X6CrNiMoTi17–12–2), differing only in the Mo content, [28]. In Ref. [29] a viscoplastic constitutive model is developed based on the results of uniaxial tensile tests on some austenitic stainless steels, also including AISI 321. In Ref. [30] the hot workability of AISI 304 and 321 austenitic stainless steels were compared through single-hit Gleeble simulated thermomechanical processing between 800 °C and 1200 °C (the strain rate varies between 0.001 s^−1^ and 5 s^−1^). An optimizing deep drilling parameter based on the Taguchi method for minimizing surface roughness was considered in Ref. [31], while the influence of nitriding time on the microstructure and microhardness of AISI 321 austenite stainless steel was investigated in Ref. [32]. The microstructural character of dissimilar welds between Incoloy 800H and 321 Stainless Steel was discussed in Ref. [33]. In Ref. [34] the effect of low temperature nitriding on the fatigue behavior of a titanium stabilized AISI 321 grade austenitic stainless steel is presented.

## 2. Information Concerning the Material, Equipment, Specimens, Test Procedures, and Standards

The material considered in this research is X6CrNiTi18-10 steel, delivered as 18 mm soft annealed round bar. The added titanium content helps in preventing chromium carbide precipitation when exposed to high temperatures, which may occur on account of wrong applications or may be generated from the welding process. The material retains good strength and is treated as very resistant to oxidation and intergranular corrosion in dry service conditions up to 850 °C. Its maximum service temperature can be significantly reduced in a hot atmosphere due to corrosive compounds such as water and sulfur compounds. However, the grade 321 possesses good creep strength. This steel is known as heat resistant steel and austenitic corrosion resistant steel. In accordance with the commonly used definition of steel as an alloy of iron and carbon (<2%) with or without the addition of other alloying elements, Table 1 presents the chemical composition of the considered steel.

The basic properties of steel depend on the chemical composition, resulting microstructure, the state, form, and dimensions of the finished product. This means that processing as a means to development and control of the microstructure as well as heat treatment are of significance for achieving the required material properties. The properties (for example: yield strength, hardness) that depend on the microstructure are called structure-sensitive properties. The general properties of this material are: good corrosion resistance, medium values of mechanical properties, average forgeability, poor machinability, excellent weldability (TIG, MAG, Arc, laser beam, and submerged arc welding, except gas welding). Core X6CrNiTi18-10 steel is titanium-stabilized steel with improved intergranular corrosion resistance and may be used in an extended temperature range. With respect to well-balanced material properties, it is suitable for many applications and can be used at elevated temperatures. As regards processing, it can be said that it is suitable for machining (but not automated), hammer and die forging, cold forming, but it is not suitable for polishing, *etc.* However, due to formation of titanium carbo-nitrides, its machinability differs from other low carbon stainless steels with titanium free variants. Its main applications are in: the automotive industry, the chemical industry (chemical processing equipment), building and construction industries, food and beverage industries, and aviation and aerospace industries (used for aircraft parts, such as exhaust systems), jet engine parts, weld equipment, power station constructions, furnace heat treated parts, oil refiners, as well as general use in mechanical engineering. Due to its vibration fatigue resistance, application is quite extensive in the aircraft industry, where operating temperatures are higher than 400 °C and where corrosive conditions are not too severe. Concerning its special property, it is usually stated to be suitable for continuous exposure to high temperatures up to 850 °C in an oxidizing atmosphere (750 °C containing sulfur). Its optimal fabrication and mechanical properties are achieved after solution annealing (temperature range: from 1075 °C to 1125 °C) followed by rapid cooling (air or water).

Equipment used in these investigations can be divided into equipment used to perform uniaxial tests at room and high temperatures and equipment used to determine impact energy. The former was the (Zwick/Roell) 400 kN capacity materials testing machine for uniaxial tests, while the latter was the Charpy impact machine. Also, if the uniaxial test was performed at room temperature then the macro extensometer was used whereas for high temperature tests high temperature extensometer and furnace (900 °C/Mytech) were used.

Specimens used in uniaxial tests to determine engineering stress-strain diagrams and in creep testing were machined from 18 mm steel rod.

The diameter of the specimen in its central part is 5 mm.

**Figure 1:**
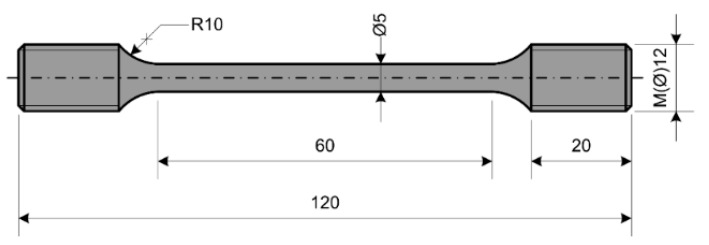
Specimen’s geometry–tensile test.

The geometry of each specimen used in the uniaxial test (room and high temperature) was defined according to that specified in ASTM: E 8M-15a, and its ends were threaded to match the holders of the testing machine, Figure 1.

Test procedure and standards in accordance with which uniaxial tests were carried out are as follows. Tensile tests related to determination of engineering stress-strain diagrams at room temperature were performed according to ASTM: E 8M-15a, while those related to high temperatures were performed in accordance with ASTM: E21-09 standard [35]. Creep testing was carried out according to the ASTM: E 139-11 standard [35]. Charpy tests were carried out according to ASTM: E23-12c standard and specimens used in these tests were also manufactured in accordance with the same standards. All of the mentioned standards can also be found in the Annual Book, Ref. [35].

## 3. Test Results and Discussion

### 3.1. Mechanical Properties-Engineering Stress-Strain Diagrams

**Figure 2:**
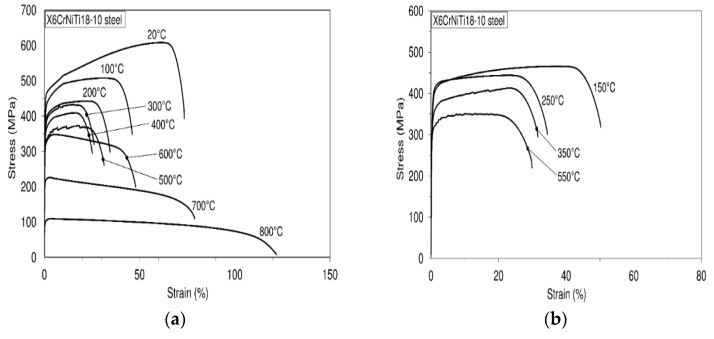
Engineering stress-strain diagrams at room and high temperatures: X6CrNiTi18-10 steel. (**a**) Stress-strain diagrams: 20 °C to 800 °C; (**b**) Stress-strain diagrams: 150 °C to 550 °C.

Engineering stress-strain diagrams were obtained by performing uniaxial tests at room and high temperatures. A minimum of five tests was performed for each test temperature. Since engineering stress-strain diagrams carried out at the considered temperature do not differ from each other at all, or a very little, only one diagram is shown for each of the considered temperatures (the first conducted test), Figure 2. On the basis of experimental investigations, the ultimate tensile strength and yield strength are said to decrease with an increase in temperature, except at a temperature of 250 °C where these properties increase a little.

The modulus of elasticity continuously decreases with an increase in temperature. Also, from the engineering stress-strain diagram, it is visible that strains at 300 °C, 400 °C, and 500 °C are somewhat reduced in comparison with the ones at higher temperatures. On the basis of engineering stress-strain diagrams it is visible that strains at 300 °C, 400 °C and 500 °C are somewhat reduced in comparison with those at higher temperatures. As a consequence of dynamic strain aging, which is treated as hardening phenomenon, in the stress-strain curves some effects can arise. Serrations in the stress-strain curves are the most visible effects of dynamic strain aging. When this effect is not seen other effects can be present. Usually, if serrations are not seen, dynamic strain aging can be marked by lower strain rate sensitivity. Also, dynamic strain aging causes a minimum variation of ductility with temperature, a plateau in strength as well as a peak in work hardening.

#### 3.1.1. Graphical Representation of the Temperature Dependency of Mechanical Properties

**Figure 3:**
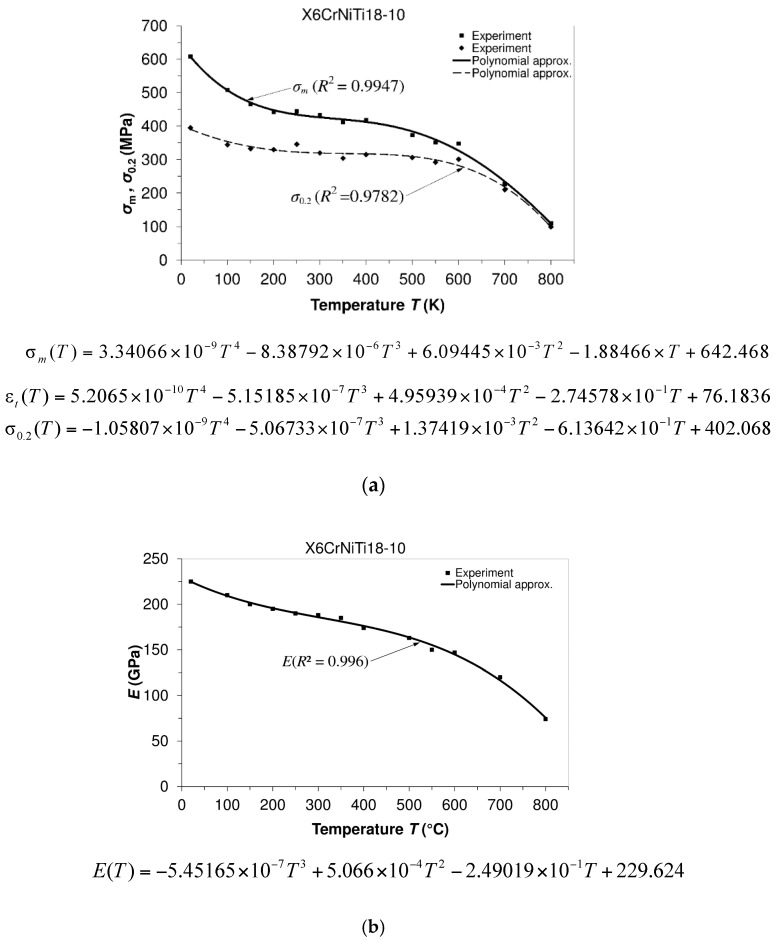

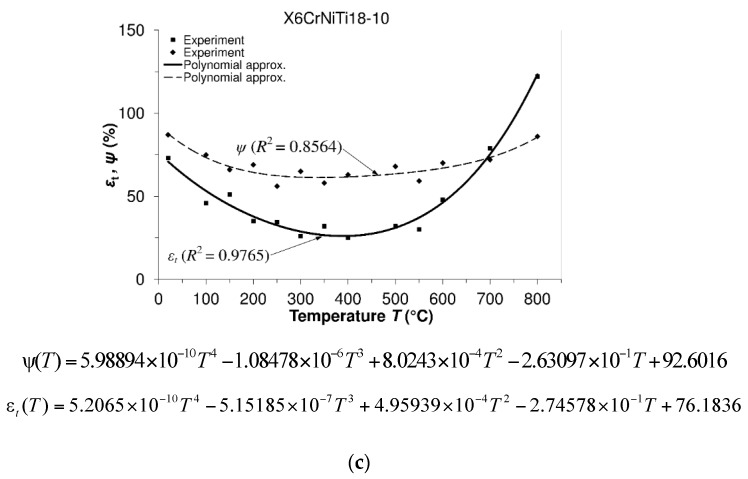
Dependence of mechanical properties on temperature: X6CrNiTi18-10 steel. (**a**) Ultimate tensile strength (σ_m_) and 0.2 offset yield strength (σ_0.2_) *versus* temperature; (**b**) The modulus of elasticity (*E*) *versus* temperature; (**c**) Total elongation (ε_t_) and reduction in the area (ψ) *versus* temperature.

Experimentally determined mechanical properties such as ultimate tensile strength, yield strength (0.2 offset yield strength), modulus of elasticity, and elongation are displayed in Figure 3 using special characters (▪, ♦). Approximation curves related to the same properties are presented using solid or dashed lines. These approximation curves relating to the mentioned properties describe their experimentally obtained values with greater or lower accuracy. To determine the accuracy of approximation, the coefficient of determination (*R*^2^) is used as a measure of accordance between experimentally obtained results and polynomial approximation and serves as a statistic that gives information on the fit of a model [36].

#### 3.1.2. Descriptive Error Bars-Mechanical Properties

**Figure 4:**
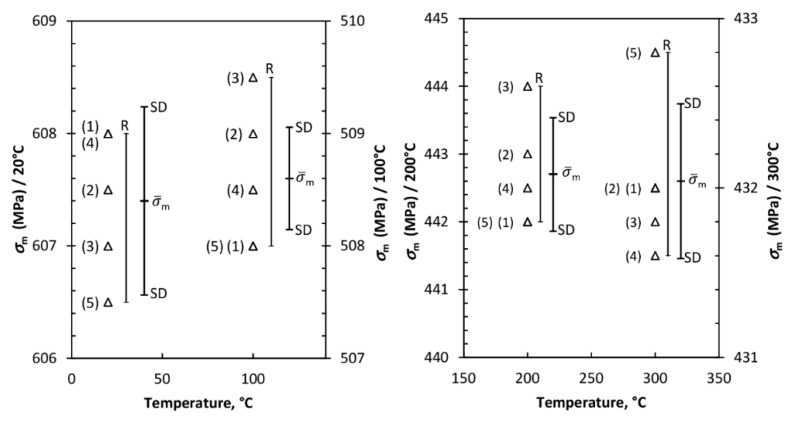

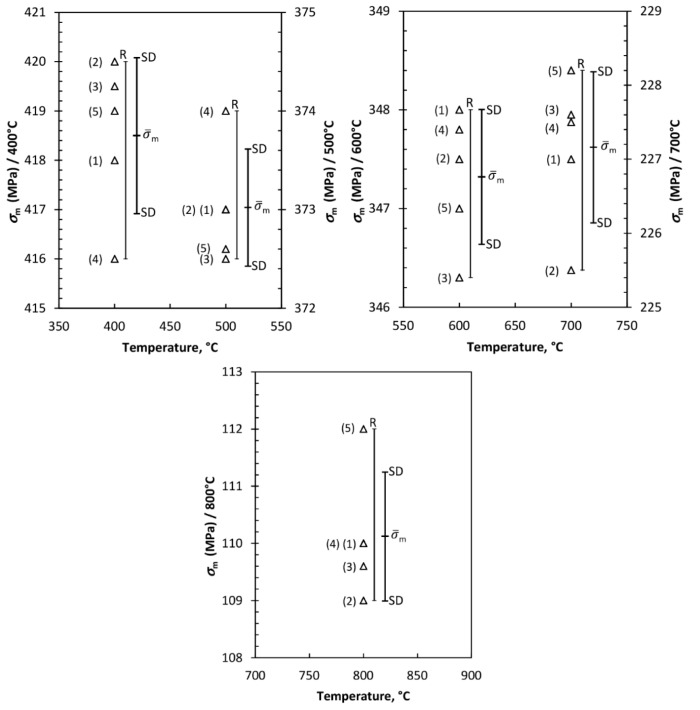
Descriptive error bars: Ultimate tensile strength of steel X6CrNiTi18-10. (The small triangles are experimental data points (test results), while *R* = range; *SD* = standard deviation; *i* = a number of the test, *n* = the total number of tests).

The structure operating under certain environmental conditions is to be subjected to certain loads. It is also necessary to choose a material whose properties will meet the structure service life requirements, *i.e.*, the ones based on known material properties so that the behavior of the structure can be predicted. Besides, very important aspects, e.g., the accuracy of the experimental results and their interpretation, arise from the experimental data. In accordance with this, several uniaxial tensile tests regarding determination of ultimate tensile strength were performed. In the so called descriptive error bars, Figure 4, an analysis of ultimate tensile strength is shown where range (*R*) and standard deviation (*SD*) are used. The standard deviation was calculated as given in Ref. [37].

(1) $$SD=\sqrt{\frac{\sum_{i}{\left(x_{i}-\bar{x}\right)}^{2}}{n-1}}$$

In Equation (1), $x_{i}$ is the individual data of the considered property at each test, $\bar{x}$ is the mean value of the considered property, calculated as:

(2) $$\bar{x}=\sum_{i}\frac{x_{i}}{n}$$

In Equation (2), n is the total number of the tests/experiments (at each test one data of the considered property is obtained). In this consideration there are: $x_{i}$ = ($\sigma_{m,i}$; $\sigma_{0.2,i}$); $\bar{x}=$(${\bar{\sigma }}_{m}$; ${\bar{\sigma }}_{0.2}$); i=1...n.

### 3.2. Determining the Resistance of Material to Creep

#### 3.2.1. Short-Time Creep Tests

**Figure 5:**
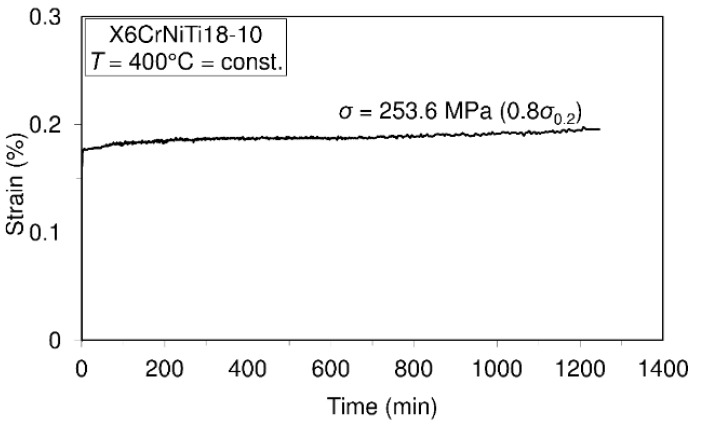
Creep behavior of steel X6CrNiTi18-10 at the temperature of 400 °C.

**Figure 6:**
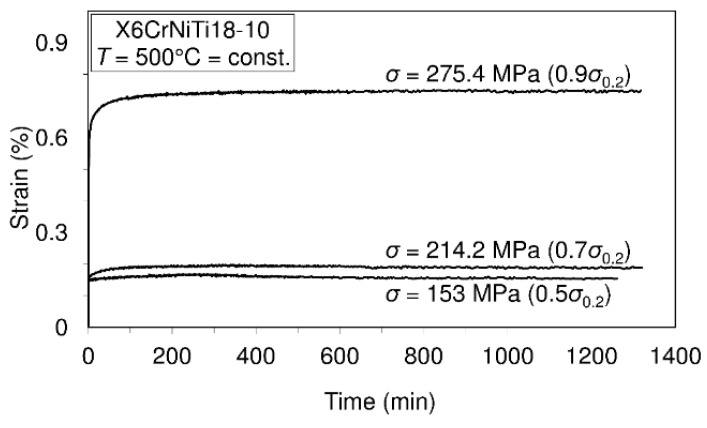
Creep behavior of steel X6CrNiTi18-10 at a temperature of 500 °C.

**Figure 7:**
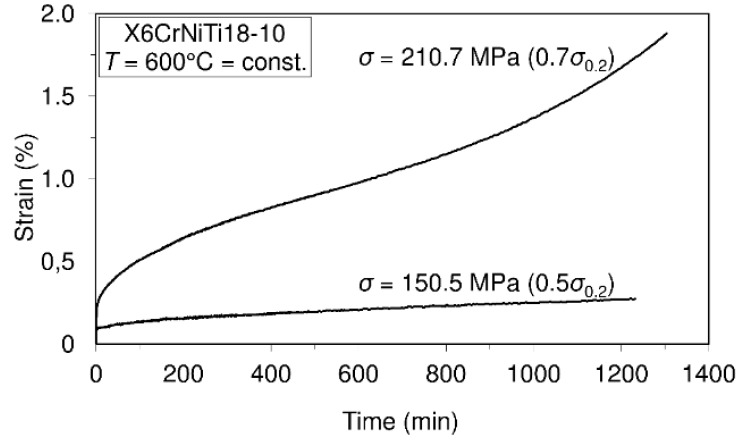
Creep behavior of steel X6CrNiTi18-10 at a temperature of 600 °C.

**Figure 8:**
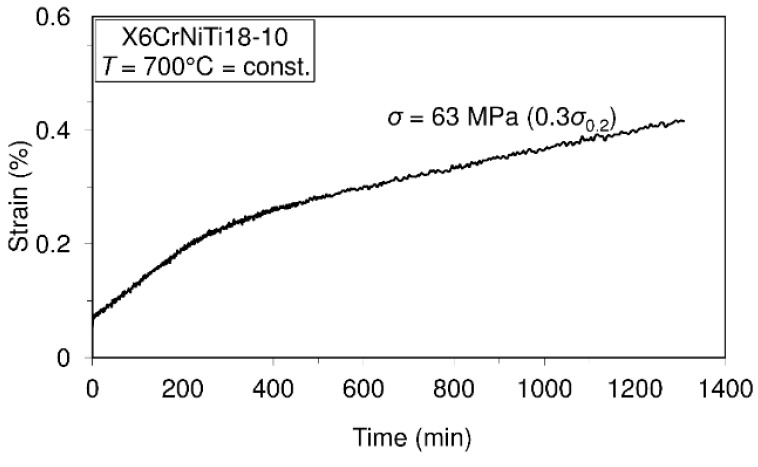
Creep behavior of steel X6CrNiTi18-10 at a temperature of 700 °C.

To assess the short-time creep behavior of the considered material or to predict its creep resistance, several uniaxial short-time creep tests were carried out. Data defining creep tests as well as material creep responses are shown in Figure 5, Figure 6, Figure 7 and Figure 8. In these investigations short-time creep behavior was considered since most materials can be subjected to such temperature conditions (occurrence of high temperature due to error in cooling, hazard, fire, *etc.*). Only some of the special materials are intended to be used in structures designed for long term operation at high temperatures. In long term creep processes, however, special equipment for creep process monitoring is to be used. In these tests, stress levels are selected in accordance with the 0.2 offset yield strength previously determined at the considered temperature (for example: 253.6 MPa is equivalent to 0.2 offset yield strength for this material at 400 °C).

In accordance with the short-time creep tests, steel X6CrNiTi18-10 is considered to be creep resistant at temperatures of 400 °C and 500 °C when the stress level does not exceed 0.9 of the yield strength at the mentioned temperatures. Also, this steel may be treated as creep resistant at a temperature of 600 °C if the applied stress does not exceed 0.5 of the yield strength at this temperature. In addition, even at temperatures of 700 °C this material may be treated as creep resistant when the applied stress does not exceed 30% of the yield strength.

#### 3.2.2. Creep Test Modeling

To perform a creep test, appropriate but usually expensive equipment is indispensable. Although the creep test shows deformation behavior of the material realistically, sometimes it is possible, based on known data of the behavior from similar conditions, to predict the creep behavior for the prescribed conditions. This prediction can be done using similar analytical methods or rheological models that are used to model/simulate real creep processes. In the following part there are two rheological models and one analytical method (formula) proposed to be used in creep modeling. All of the proposed tools can be used for modeling the first and second creep stages. Well known rheological models are the Burgers model and the Standard linear solid model (SLS). The Burgers model is represented in Refs. [20,38]:

(3) $$\varepsilon \left(t\right)=\sigma \left[\frac{1}{E_{1}}+\frac{1}{E_{2}}\left(1-e^{\left(-E_{2}/\eta_{1}\right)t}\right)+\frac{t}{\eta_{2}}\right]$$

In Equation (3) there are: ε(t)*-*strain, σ-stress, *E*_1_*-*modulus of elasticity, *t-*time, all related to considered creep curve, while $E_{2}$, $\eta_{1}$, and $\eta_{2}$ are parameters whose values are determined on the basis of equality of the discussed creep curve and Equation (3). The Standard linear solid model (SLS) is represented Refs. [22,39]:

(4) $$\varepsilon \left(t\right)=\frac{\sigma }{E_{1}}+\sigma \left(\frac{1}{E_{1}+E_{2}}-\frac{1}{E_{1}}\right)e^{-\frac{E_{1}E_{2}t}{\left(E_{1}+E_{2}\right)\eta }}$$

In Equation (4) there are: ε(t)-strain, σ-stress, *t*-time, *E*_1_, *E*_2_, and η are parameters whose values are determined on the basis of equality of the discussed creep curve and Equation (4).

An analytical formula to model/simulate creep behavior is proposed in Refs. [11,12,22]:

(5) $$\varepsilon \left(t\right)=D^{-T}\sigma^{p}t^{r}{}^{}$$

In Equation (5) there are: *T-*temperature, σ-stress, *t-*time and *D*, *p* and *r* are parameters which are to be determined. All of the mentioned Equations (3)–(5) can be used for three different types of modelling, namely, for:

(6a) ε(t)=ε(t), σ=const, T=const

(6b) ε(t)=ε(σ, t), T=const

(6c) ε(t)=ε(σ,T,t)

**Figure 9:**
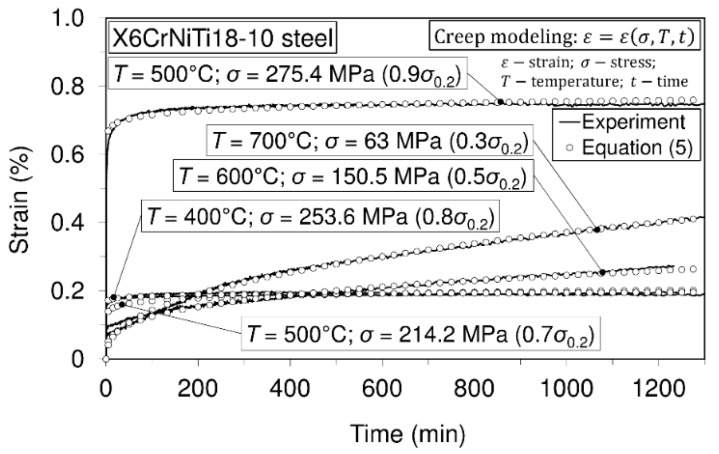
Experimental and modeled creep curves: steel X6CrNiTi18-10. Modeling was performed using Equation (5) by applying the principle 6c.

The first type of modeling, Equation (6a), denotes the modeling of one exactly defined creep curve that is described by the defined creep temperature and the defined stress level at this temperature. The last type of modeling, Equation (6c), is the most useful and applicable one. Namely, this modeling covers an entire range of stress levels and temperature levels for the considered time range. In Table 2, data relating to creep modelling are presented, while creep modelling curves are presented in Figure 9.

In general, all of the above mentioned models for simulating creep behavior are considered to be satisfactory. However, an analytical formula is proposed as the best model. When rheological models are considered, then the Burgers model seems to be more suitable for the creep processes where the dominated creep phase is the steady-state phase, while for creep process with dominated transient phase (more expressed parabolic shape), the SLS model is considered to be more suitable. Also, it needs to be said that any of the used models are more suitable when only a particular curve is selected to be modeled.

### 3.3. Correlation between Charpy V-Notch Impact Energy and Fracture Toughness

Undoubtedly fracture toughness is one of the most important material properties used to design structure against fracture. Fracture toughness is a parameter that defines material resistance to crack extension [40], and it represents a critical value of the stress intensity factor (*SIF* or *K*), designated as *K*_Ic_. Designation $K_{\text{Ic}}$ indicates that this is a minimum value of fracture toughness determined by using a specimen that meets the plane strain conditions and besides, is tensile loaded (first mode). Fracture toughness is stated to be a subject concerned with predicting failure mode, especially one containing crack-like defects [41]. In engineering practice, fracture toughness can be experimentally determined by a number of standard tests [42]. However, experiments related to the measurement of fracture toughness are not intended for materials that behave plastically. In this case, the *J*-integral (or, for example, CTOD) as a fracture parameter is fairly appropriate. On the other hand, to avoid problems such as complicated manufacturing of the specimen, differences between the crack in the real structure, and the manufactured crack on the specimen, other methods existing for fracture toughness assessment are used. One of these such methods is the determination of the Charpy impact energy. Correlation between the Charpy impact energy and fracture toughness was reviewed in Refs. [43,44]. In these investigations the Charpy test was used to assess the fracture toughness. The specimens were machined from the material rod (longitudinal direction). The formula used to assess fracture toughness is based on the measured Charpy V-notch impact energy, *i.e.*, it is a well-known Roberts-Newton formula that is independent of the temperature:

(7) *K*_Ic_ = 8.47 (*CVN*)^0.63^

**Figure 10:**
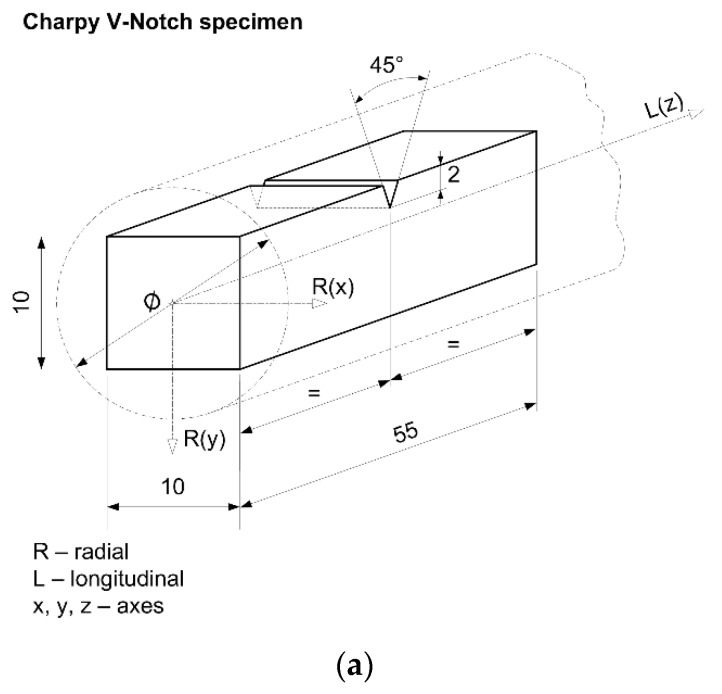

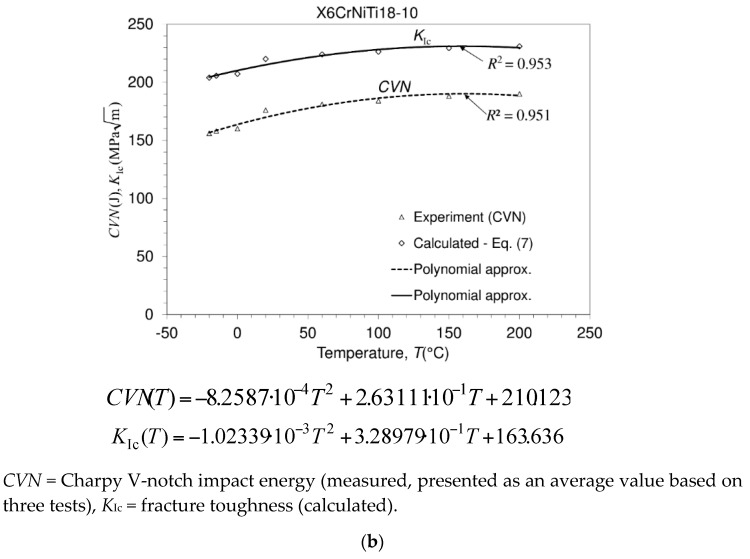
Charpy V-notch specimen and impact energy determination. (**a**) Specimen’s geometry-Charpy V-notch impact energy determination; (**b**) Charpy V-notch energy (CVN); and calculated fracture toughness (*K*_Ic_) *versus* temperature.

However, although this formula can be applied independently of the temperature, the *CVN* impact energy is inserted in it in accordance with the obtained result at the considered temperature. The geometry of the specimens used in these investigations is shown in Figure 10a. Experimentally obtained results of the Charpy V-notch impact energy and the calculated values of fracture toughness are presented in Figure 10b. Note: Specimen (10 × 10 × 55 mm^3^) is manufactured from a rod in such way that its largest dimension (55 mm) coincides with the length of the rod.

### 3.4. Fatigue Testing of Tensile Stressed Specimens

#### 3.4.1. General Consideration

As engineering structures are frequently subjected to different loadings, it is surely of interest to investigate the resistance of the material to failure at the mentioned loadings. It is known that when a material is subjected to a repeated load (cyclic load), fatigue failure may result in fracture of the considered engineering element at a stress level that is much lower than the fracture stress corresponding to a monotonic tensile load. Fatigue may be defined as the process of damage accumulation due to cyclic loading [45]. The main task for the designer dealing with a structure design subjected to repeated load is to ensure the required structure service life. Fatigue design criteria are based on known fatigue life models in accordance with the required durability of the structure. These models are: stress-life model, deformation-life model and crack-propagation rate model. In this paper a stress-life model is applied. In these investigations the specimens were subjected to tensile stresses at the prescribed stress ratio and the cyclic loading processes were carried out in accordance with the sinusoidal law. The abscissa (the number of cycles (*N*) to failure) is usually plotted on a logarithmic scale, while the ordinate (cyclic maximum stress, or cyclic mean stress or stress amplitude) is plotted on a linear scale or on a logarithmic scale. Fatigue can be considered in the cases of tensile loading (or another type of load) for unnotched (smooth) or notched specimens. However, attention should be paid to ensure that the test specimen is compatible with the testing structure generating test data. The geometry (including dimensions) of the specimen as well as the testing procedure are recommended by standard (ASME standard, ISO standard). Each fatigue test performed at the prescribed fatigue stress and at the prescribed stress ratio generates one point in the *S-N* system. Usually several fatigue tests are performed for the same fatigue stress level since a scatter related to the number of cycles to failure exists. On the basis of the recorded test data the *S-N* system, or *S-N* diagram (also called *S-N* curve, Wöhler curve or fatigue life) is created and it represents the applied fatigue stresses *versus* the number of cycles to failure for the considered material and for the prescribed stress ratio. *A*. Wöhler, a German scientist [46] carried out numerous tests using smooth and notched specimens taken from railway axles. When regardless of the number of cycles the test specimen remains unbroken (*i.e.*, without the occurrence of failure) at the prescribed conditions of the test, the fatigue limit has been reached, and the stress associated with this limit is called the fatigue limit (endurance limit) [3,47,48]. This in fact means that there is a theoretical level of stress for the prescribed stress ratio below which the material will not fail for any number of cycles. The *S-N* diagram consists of two parts (areas) of which the first area belongs to the finite fatigue region (finite fatigue life) and the second one that belongs to the infinite fatigue regime (life). If both parts are presented in linear form, then the diagram contains the inclined and the horizontal line. Namely, in this case the infinite part of the *S-N* curve (infinite fatigue region) becomes practically horizontal. This is valid for a material with clearly expresses fatigue limit. By contrast, the *S-N* curve may have a shape that corresponds to the material without clear fatigue limit. In the standards as well as in literature in general, a proposition related to the performing of the fatigue test and also for fatigue limit determination can be found. For steel alloys, as fatigue limit, the number of the cycles at which the specimen remains unbroken, is usually adopted in an amount of 10^7^ cycles. Some engineering components are subjected to low stresses relative to the material ultimate tensile strength but at a very large number of cycles and that due to long service life and/or high frequency load. So, high cycle fatigue (HCF) is treated as crack growth phenomenon related to the previously mentioned stressed components. In any case, in engineering practice the influence of HCF exerted on the life of the component is to be eliminated or reduced to a minimum. If testing procedures require 10^9^ cycles, it may be said that these cases belong to the so called ultra-high cycle fatigue regime (UHCF). Some factors such as surface finish, applied loading conditions, deviations in specimen alignment, *etc.*, cause scatter in fatigue data. A group of methods also exists that provides a means to deal with the scatter fatigue data and in this sense provide an estimate for median fatigue strength at the considered number of cycles. To the mentioned group of methods (tools) there may be counted: conventional *S-N* tests, advanced statistical methods, quantal response tests, and accelerated stress tests [45]. Within these groups there are several methods: for example, methods such as the Staircase method, Two-point method, *etc.* which belong to the quantal response tests (regarded as a group of methods). Conversely, fatigue limit is often considered to be a material property.

#### 3.4.2. Fatigue Testing

**Figure 11:**
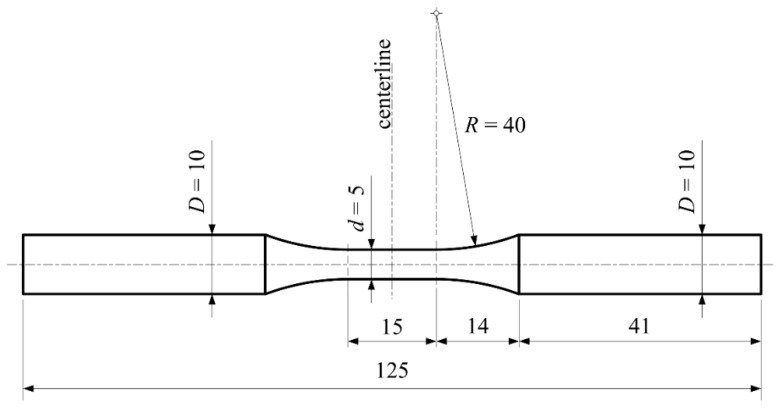
Specimen’s geometry-fatigue test.

**Figure 12:**
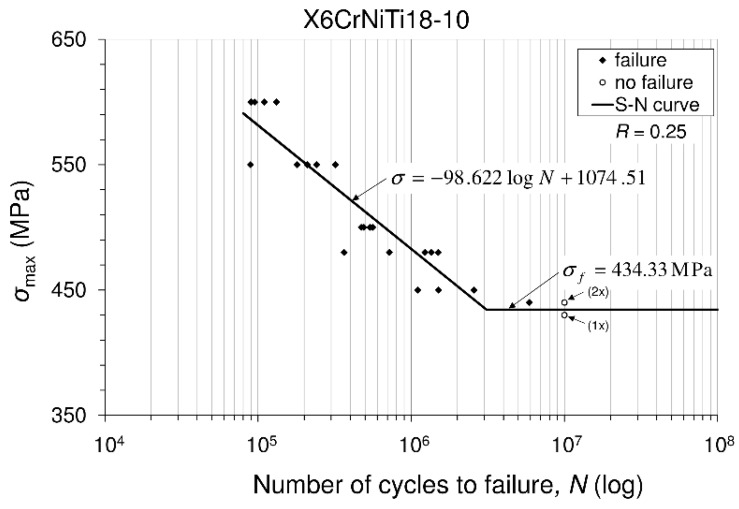
Fatigue tests (at room temperature and stress ratio *R* = 0.25) for X6CrNiTi18-10 steel: maximum stress *versus* number of cycles to failure (○ = no failure, ♦ = failure), approximated “*S-N*” curve (line) in fatigue limit region (\) and fatigue limit (endurance limit) in fatigue infinite region (–).

In the case presented in this paper, fatigue tests of cyclic tensile stressed specimens were carried out in accordance with the standard ISO 12107:2012 (2012) [49], at the prescribed stress ratio of *R* = 0.25 and at room temperature. The geometry of the specimen is shown in Figure 11 while test results data as well as fatigue limit are presented in Figure 12.

Fatigue testing started in such a way that the first tests were at the stress level (600 MPa) near the tensile strength (608 MPa) of the tested material. Afterwards these specimens were tested by decreasing stress regime. A number of 10 million cycles was selected as the desired number of cycles at which the specimen remains unbroken. In general, at the considered stress level some specimens may be expected to be broken (failed) while some others may not. Namely, in some cases there is a runout where the time to failure exceeds that available for the test (censoring). As is visible, in this stress-life model, the entire procedure of fatigue testing included 21 tests of which seven tests belong to the staircase method (modified staircase method) used for fatigue limit determination. In finite fatigue life the approximated curve (line) is determined without taking into account runouts.

#### 3.4.3. Fatigue Limit Calculation

The fatigue limit (endurance limit) or so called fatigue strength in the infinite fatigue life range can be estimated in accordance with the modified staircase method and in Figure 12 it is presented by the horizontal line. Specimens were tested under decreasing stress regime. In Table 3 data related to failed and not-failed specimens for analysis in the modified Staircase method are presented. Fatigue data used in the modified Staircase method analysis are completed from fatigue testing. Data are presented using the following designations: specimens failed (♦) or not-failed (○) for the appropriate stress levels at a certain number of cycles. In this sense, fatigue stress levels applied are: 450 MPa (as $\sigma_{2}$, the last stress level from the finite fatigue life, hence it is visible that all of the specimens failed), 440 MPa (as $\sigma_{1},$ first stress level at which the first and last specimens occur that are not-failed among one that failed), 430 MPa (as $\sigma_{0}$, stress level at which specimen not-failed). It is worth pointing out that the highest level of the stress was $\sigma_{2}$ and it coincides with the lowest stress measured in the finite fatigue regime. Let, in general, the magnitude of stress be designated as “$\sigma_{i}$” (MPa) related to the specific “i-th” level of testing, and the number of failure events as “*f_i_*” related to the same specific (considered) stress level. As observed, each stress level (430, 440, 450) increases to the previous one in stress intensity which is equal to the stress step, *d* = 10 MPa. Table 4 presents an analysis of the staircase data needed for the derivation of constants. Furthermore, the mentioned constants are needed for the fatigue limit calculation, Table 5.

The lower limit of fatigue strength in infinite fatigue life (*i.e.*, fatigue limit, endurance limit) is defined as (in accordance with ISO standard):

(8) $$\sigma_{f\left(P,\;1-\alpha \right)}={\bar{\mu }}_{y}-k_{\left(P,1-\alpha ,dof\right)}\times {\bar{\sigma }}_{y}$$

In this Equation there are: The mean fatigue strength

(9) $${\bar{\mu }}_{y}=\sigma_{0}+d\left(\frac{A}{C}-\frac{1}{2}\right)$$

where *d* is stress step-The coefficient for the one sided tolerance limit for a normal distribution $k_{\left(P,1-\alpha ,\nu \right).}$ The estimated standard deviation of the fatigue strength

(10) $${\bar{\sigma }}_{y}=1.62\;\times \;d\left(D\;+\;0.029\right).$$

In accordance with the standard recommendation of ν =n − 1= 6 where *n* is the number of items in a considered group. Consecutively, for a desired probability of P=10% and a confidence level (1−α) = 90%, in accordance with the table B_1_ (ISO), it follows:$\;k_{\left(P,1-\alpha ,\nu \right)}\;$= $k_{\left(0.1;0.9;6\right)}=\;$2.333. In accordance with Equations (9) and (10), it is:

(11) $${\bar{\mu }}_{y}=\sigma_{0}+d(\frac{A}{C}-\frac{1}{2})=430+10(\frac{7}{4}\times \frac{1}{2})=442.5\;\text{MPa}$$

or this can be obtained as:

(12) $${\bar{\mu }}_{y}=\frac{430+440+450+440+450+440+450}{7}=442.8$$

(13) $${\bar{\sigma }}_{y}=1.62\;\times d\left(D+0.029\right)=1.62\times 10(0.1875+0.029)=3.5\;\text{MPa}$$

Finally, the fatigue limit is:

(14) $$\sigma_{f\left(0.1;0.9;6\right)}={\bar{\mu }}_{y}-k_{\left(P,1-\alpha ,\nu \right)}\;\times \;{\bar{\sigma }}_{y}=442.5-2.333\times 3.5=434.33\;\text{MPa}$$

Similar investigations related to fatigue of X6CrNiTi18-10 steel have not been found in the literature so far. However, many materials have been tested for fatigue as well as phenomena that occur with fatigue such as those considered in [50].

### 3.5. Basic Analysis of the Microstructure

**Figure 13:**
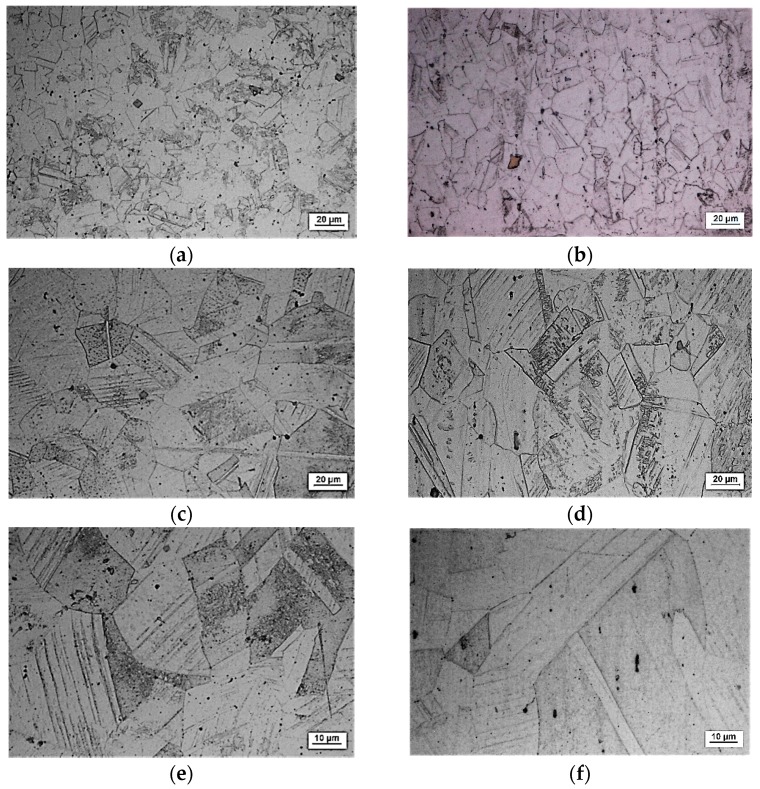
X6CrNiTi18-10 steel: optical micrographs, aqua regia. (**a**) As-received material (austenitic stainless steel, annealed bar), the cross-section of the specimen, 500×; (**b**) As received material, the longitudinal-section of the specimen, 500×; (**c**) After performing the creep process at 500 °C/153 MPa/1200 min, the cross-section of the specimen, 500×; (**d**) After performing the creep process at 500 °C/153 MPa/1200 min, the longitudinal-section of the specimen, 500×; (**e**) After performing the creep process at 700 °C/63 MPa/1200 min, the cross-section of the specimen, 1000×; (**f**) After the creep process performed at 700 °C/63 MPa/1200 min, the longitudinal-section of the specimen, 1000×.

As is well known, material properties depend on the chemical composition, processing path, and microstructure. For the considered steel and its composition, some of properties depend on the microstructure. The properties that depend on the microstructure are called structure sensitive properties (for example, yield strength, hardness). Cold or hot rolling, for example, is the means to develop and control the microstructure. Since the microstructure can be changed when the material is exposed to high temperature, three specimens exposed to different temperature conditions were examined. The first specimen is referred to as delivered material, and the other two specimens with reference to creep conditions carried out at different temperatures and different stress levels. The former creep test was carried out at 500 °C, 153 MPa, 1200 min and the latter one at 700 °C, 63 MPa, 1200 min. Optical micrographs of the examined specimens are shown in Figure 13.

On the basis of the given micrographs, it can be said as follows. The basic microstructure (main phase/main structure) of as-received material is austenite characterized by polygonal austenite grains, Figure 13a,b. Also, there is a mixture of austenite and ferrite. A lot of impurity exists in the material and this is distributed in the form of non-uniform black particles. In between there are twin grains. Considering the longitudinal section, it can be concluded that the slip line and twin crystals are included in the austenite. The ferrite has been stretched and that suggests that the material has suffered some plastic deformation in the longitudinal direction. In the case of the specimen that was previously subjected to creep (500 °C/153 MPa/1200 min), Figure 13c,d, the temperature of the creep process was not so high but the stress level was high and the austenite grains were increased. It is shown that not only slip lines increase after creep deformation, but the ferrite content increases as well. Some ferrite precipitated from the austenite which indicated that the stability of the austenite of this material was not good enough. At the same time a higher number of twin grains occurs which have better polygonal shapes. Also, the formation of holes due to the diffusion of failures in the crystal lattice is noticed. As regards the case presented in Figure 13e,f; that is, when the specimen was previously subjected to creep (700 °C/63 MPa/1200 min), since the temperature increases, although the stress level was low the temperature increased, the grains are increased and the number of twin grains also increases. In this case, the influence of high temperature on the deformation process is apparent. Some recrystallized grains can be found, which indicates that this material starts to recrystallize at a temperature of 700 °C.

## 4. Conclusions

The results of this research present very good information for designers involved in design of structures made of steel X6CrNiTi18-10. The obtained results relating to tensile strength and yield strength show that this material has good ultimate tensile strength and yield strength not only at room temperature but also at high temperatures as is apparent from Table 2. Furthermore, the steel is creep resistant over a wide range of temperatures and stress levels, as can be seen from Figure 5, Figure 6, Figure 7 and Figure 8. Also, after carrying out the tests, the fatigue limit of the cyclic tensile stressed steel at a stress ratio of *R* = 0.25 can be adopted in an amount of 434.33 MPa. Finally, the steel is characterized by an impact energy obtained with the Charpy impact machine in an amount of 176 J/20 °C from which a fracture toughness of $220\;\text{MPa}\;\sqrt{m}$ was calculated.

## Figures and Tables

**Table 1:** Material chemical composition: X6CrNiTi18-10 steel.

Material: X6CrNiTi18-10								
Designation								
Steel name				Steel number (Mat. No; W. Nr; Mat. Code)				
EN 10088-3-2014/DIN 17440: X6CrNiTi18-10; AISI 321; ASTM ~ A249 (321) UNS S32100; BS: 321S51.				1.4541				
Chemical composition of considered material Mass (%)								
C	Si	Mn	P	S	Cr		Ni	V
0.0176	0.436	1.44	0.0209	0.0174	16.95		9.263	0.204
Mo	Cu	Al	Ti	Pb	Sn	Co	W	Rest
0.241	0.548	0.0208	0.266	0.006	0.0443	0.1	0.107	70.318

**Table 2:** Creep modeling data: X6CrNiTi18-10 steel.

Material: X6CrNiTi18-10 steel				
Application of Equation (6c) ε = $\varepsilon (\sigma ,T,t)=D{\left(T\right)}^{-T}\cdot \sigma^{p(T)}\cdot t^{r(T)}$				
Constant temperature *T* (°C)	400	500	600	700
Constant stress level σ(MPa) = *x* ×$\;\sigma_{0.2}$	*x* ≤ 0.9 376	*x* = 0.7–0.9 214–275	*x* ≤ 0.5 150	*x* ≤ 0.3 63
Time (min)	1200			
Analytical Equation (5)	Parameters			
	$D(T)=6.150138\times 10^{-8}T^{3}-1.1354401\times 10^{-4}T^{2}+6.7433968\times 10^{-2}T-11.784261$ $p(T)=7.7811369\times 10^{-7}T^{3}-1.6357754\times 10^{-3}T^{2}+1.0613631\times T-213.60664$ $r(T)=-7.751213\times 10^{-8}T^{3}+1.3200375\times 10^{-4}T^{2}-7.1958106\times 10^{-2}T+12.690118$			

**Table 3:** Data for modified staircase method related to failed (♦) and not-failed (○) specimens.

Stress $\sigma_{i}\;$MPa	Number of Specimen						
	1	2	3	4	5	6	7
450	–	–	●	–	●	–	●
440	–	○	–	●	–	○	–
430	○	–	–	–	–	–	–

**Table 4:** Data analysis for modified staircase method.

Stress $\sigma_{i}\;$(MPa)	Level of Stress *i*	*f*_i_	*if*_i_	*i*^2^*f*_i_
450	2	3	6	12
440	1	1	1	1
430	0	0	0	0
–	$\sum^{}$	4	7	13

**Table 5:** Calculation of the constants *A*, *B*, *C*, *D* in accordance with ISO standard.

Formula	Material: X6CrNiTi18-10
$A=\sum^{}i\times f_{i}$	7
$B=\sum^{}i^{2}\times f_{i}$	13
$C=\sum^{\text{​}}f_{i}$	4
$D=\frac{B\times C-A^{2}}{C^{2}}$	0.1875
